# Risk of failing both methotrexate and mycophenolate mofetil from the First-line Antimetabolites as Steroid-sparing Treatment (FAST) uveitis trial

**DOI:** 10.1186/s12348-023-00350-5

**Published:** 2023-06-09

**Authors:** Amit K. Reddy, D. Claire Miller, Amol A. Sura, SR Rathinam, John A Gonzales, Radhika Thundikandy, Anuradha Kanakath, Bala Murugan, Rajesh Vedhanayaki, Lyndell L. Lim, Eric B. Suhler, Thuy Doan, Hassan A. Al-Dhibi, Debra A. Goldstein, Lourdes Arellanes-Garcia, Nisha R Acharya

**Affiliations:** 1 F.I. Proctor Foundation, University of California, San Francisco, 490 Illinois St Fl 2, San Francisco, CA 94158 USA; 2 Department of Ophthalmology, University of California, San Francisco, San Francisco, CA USA; 3 Aravind Eye Hospitals and Postgraduate Institute of Ophthalmology, Uvea Services, Madurai, India; 4 Aravind Eye Hospitals and Postgraduate Institute of Ophthalmology, Uvea Services, Coimbatore, India; 5 Aravind Eye Hospitals and Postgraduate Institute of Ophthalmology, Uvea Services, Pondicherry, India; 6 Centre for Eye Research Australia, Royal Victorian Eye and Ear Hospital, East Melbourne, Australia; 7 Casey Eye Institute, Oregon Health and Science University, Potland, OR USA; 8 Division of Vitreoretinal Surgery and Uveitis, King Khaled Eye Specialist Hospital, Riyadh, Kingdom of Saudi Arabia; 9 Department of Ophthalmology, Northwestern University Feinberg School of Medicine, Chicago, IL USA; 10 Asociación Para Evitar la Ceguera, Mexico City, Mexico; 11 Department of Epidemiology and Biostatics, University of California, San Francisco, San Francisco, CA USA

**Keywords:** Non-infectious uveitis, Immunomodulatory therapy, Methotrexate, Mycophenolate mofetil, Retinal vasculitis

## Abstract

**Background:**

The antimetabolites methotrexate (MTX) and mycophenolate mofetil (MMF) are commonly used as initial corticosteroid-sparing treatment for uveitis. There is little data examining risk factors for failing both MTX and MMF. The objective of this study is to determine risk factors for failing both MTX and MMF in patients with non-infectious uveitis.

**Main body:**

This is a sub-analysis of the First-line Antimetabolites as Steroid-sparing Treatment (FAST) uveitis trial, which was an international, multicenter, block-randomized, observer-masked, comparative effectiveness trial comparing MTX and MMF as initial treatments for non-infectious uveitis. This study was undertaken at multiple referral centers in India, the United States, Australia, Saudi Arabia and Mexico between 2013 and 2017. A total of 137 patients who completed all 12 months of follow-up from the FAST trial, were included in this study. The primary outcome was failing both antimetabolites over the 12 months of the trial. Potential predictors included: age, sex, bilateral involvement, anatomic location of the uveitis, presence of cystoid macular edema (CME) and retinal vasculitis at baseline visit, uveitis duration, and country/study sites as risk factors for failing both MTX and MMF. The presence of retinal vasculitis posterior to the equator on fluorescein angiogram was associated with failing both MTX and MMF.

**Conclusion:**

Retinal vasculitis may be a risk factor for failing multiple antimetabolites. Clinicians could consider more quickly advancing these patients to other medication classes, such as biologics.

## Background

Immunosuppressive therapy is a mainstay in the treatment of uveitis [1], with the aim of controlling ocular inflammation while avoiding the long-term side effects of local and systemic corticosteroid use. The antimetabolites methotrexate (MTX) and mycophenolate mofetil (MMF) have both been shown to be effective in managing uveitis [2, 3] and are commonly used as initial corticosteroid-sparing treatment for uveitis. However, a significant percentage of patients will have disease that is refractory to one antimetabolite therapy [4]. In these situations, it is often not clear if another antimetabolite should be tried, or if the patient should be advanced to other classes of immunosuppressive medications, such as biologic therapies, which are generally more expensive and can be associated with serious adverse effects [5]. There is limited information on the utility of switching antimetabolites after failing one. One retrospective study found that patients with scleritis and juvenile idiopathic arthritis-associated uveitis were more likely to fail MMF after previously failing MTX [6]. No prior studies have evaluated this question using prospective data.

The First-line Antimetabolites as Steroid-sparing Treatment (FAST) uveitis trial compared MTX and MMF as initial treatments for noninfectious uveitis [7]. In this study, we conducted a sub-analysis of the FAST trial to identify risk factors for failing both MTX and MMF.

## Methods

The FAST trial was an international, multicenter, block randomized, observer-masked, comparative effectiveness trial. The trial’s full methodologic details and primary study outcomes have been previously published [7]. Briefly, patients were initially randomized to either MTX or MMF, with a standardized oral prednisone taper beginning with the lesser of 60 mg or 1 mg/kg daily [7]. There was no difference in corticosteroid exposure between the two treatment groups in terms of baseline or total corticosteroid exposure during the study [8]. The primary outcome of the main trial was corticosteroid-sparing control of inflammation in both eyes at 6 months, with MMF not found to be superior to MTX in this regard. Patients who were considered treatment successes continued to take the same medication for another 6 months, while treatment failures were switched to the other antimetabolite for the remaining 6 months. Treatment failure could be declared due to efficacy, safety, or tolerability.

This sub-analysis evaluated the patients who completed all 12 months of follow-up. The main outcome was comparing patients who succeeded on either antimetabolite to patients who failed both antimetabolites over the 12 months of the trial. Age at enrollment, sex, bilateral involvement, anatomic location of the uveitis, presence of cystoid macular edema (CME) and retinal vasculitis at the baseline visit, uveitis duration, and country/study sites were evaluated as potential risk factors. Ocular coherence tomography (OCT) scans were graded in a masked fashion at the reading center at the University of South Florida. Patients with sub-retinal fluid in the setting of Vogt-Koyanagi-Harada disease were not included as having macular edema. Retinal vasculitis was defined as vascular leakage posterior to the equator on fluorescein angiogram (FA). While OCT imaging was obtained at all visits, FA was done at the discretion of the treating physicians.

### Statistical analysis

Descriptive statistics were calculated for the baseline variables of interest above. Univariate associations with failing both antimetabolites were evaluated using t-tests for continuous variables and chi-square tests or Fisher’s exact tests for categorical variables. A logistic regression model was fit to the data to model the odds of failing both MTX and MMF. Stepwise selection based on Akaike information criterion using both backward and forward selection was used to identify the final logistic regression model. Variables with significant associations with double treatment failure in the univariate analyses (*p* < 0.05) were required to stay in the model. Due to the relatively large number of patients without FA images, further sensitivity analysis was performed by simulating various scenarios of retinal vasculitis prevalence in those patients without FA images. Random intercepts for site and country were tested, but due to the small sample size, no random effects could be included in the final model. Statistical analyses were performed in R version 4.0.3.

## Results

Out of 216 patients randomized in the FAST trial, 163 patients (76%) completed all 12 months of follow-up. 26 patients were considered treatment successes for the first 6 months, continued on the same antimetabolite, and then failed at 12 months. These patients were not included in this study, leaving 137 patients for evaluation. Of these, 115 patients were considered treatment successes at 12 months - 88 patients who were kept on the same antimetabolite throughout the study and 27 patients who were treatment failures at 6 months on one antimetabolite, but then controlled at 12 months on the other antimetabolite. Of these 27 patients, only three had retinal vasculitis defined by vascular leakage posterior to the equator on FA. The 22 remaining patients failed both MTX and MMF (failed one antimetabolite at 6 months, then the other antimetabolite at 12 months).

Of these 137 patients, 67% (92) were female and the mean age was 40 years. Seventy-six patients (56%) had FA images obtained at the baseline visits. Age, sex, bilateral involvement, uveitis duration, and country/study sites were not associated with failing both antimetabolites in the univariate analysis. Conversely, failing both antimetabolites was associated with a classification of anterior/intermediate uveitis, the presence of CME, and the presence of retinal vasculitis in the univariate analysis (Table 1).

**Table 1:** Characteristics of treatment successes and double treatment failures

**Baseline characteristic**	**Success (*N*=115)**	**Double Failure (*N*=22)**	**All (*N*=137)**	***P*-value**
**Sex**				
Female	76 (66%)	16 (73%)	92 (67%)	0.72
Male	39 (34%)	6 (27%)	45 (33%)	
**Age (years)**				
Mean (SD)	41 (15)	35 (17)	40 (15)	0.14
Median (Q1, Q3)	39 (30, 53)	29 (23, 46)	37 (26, 53)	
**Laterality**				
Unilateral	10 (8.7%)	3 (14%)	13 (9.5%)	0.44
Bilateral	105 (91%)	19 (86%)	124 (91%)	
**Anatomic location**				
Anterior/Intermediate	18 (26%)	10 (46%)	28 (20%)	0.003
Posterior/Panuveitis	97 (74%)	12 (55%)	109 (80%)	
**CME**				
No	85 (74%)	10 (46%)	95 (69%)	0.016
Yes	30 (26%)	12 (55%)	42 (31%)	
**Retinal vasculitis**				
No	58 (50%)	4 (18%)	62 (45%)	0.006
Yes	9 (7.8%)	5 (23%)	14 (10%)	
FA not done	48 (42%)	13 (59%)	61 (45%)	
**Uveitis duration (months)**				
Mean (SD)	25 (52)	31 (52)	26 (52)	0.63
Median (Q1, Q3)	3.2 (0.63, 25)	7.9 (3.5, 27)	3.8 (0.64, 26)	
Missing	1 (0.9%)	1 (4.5%)	2 (1.5%)	
**Country**				
Australia	8 (7.0%)	4 (18%)	12 (8.8%)	0.18
India	77 (67%)	12 (55%)	89 (65%)	
Saudi Arabia	7 (6.1%)	0 (0%)	7 (5.1%)	
US/Mexico	23 (20%)	6 (27%)	29 (21%)	
**Study site**				
Coimbatore	21 (18%)	4 (18%)	25 (18%)	0.18
Madurai	35 (30%)	6 (27%)	41 (30%)	
Melbourne	8 (7.0%)	4 (18%)	12 (8.8%)	
Mexico	1 (0.9%)	0 (0%)	1 (0.7%)	
Pondicherry	21 (18%)	2 (9.1%)	23 (17%)	
Portland	5 (4.3%)	4 (18%)	9 (6.6%)	
Riyadh	7 (6.1%)	0 (0%)	7 (5.1%)	
San Francisco	17 (15%)	2 (9.1%)	19 (14%)	

The final model included age, sex, anatomic location, CME, and retinal vasculitis as covariates. Within the final model – after adjusting for age, sex, anatomic location, and the presence of CME – the presence of retinal vasculitis was the only characteristic that remained statistically significantly associated with failing both antimetabolites (adjusted odds ratio [OR], 8.6 [95% CI, 1.6 to 47]; *P* = 0.014) (Table 2). In further sensitivity analysis, this result remained statistically significant in simulations of different prevalence of retinal vasculitis in those without FA images, even in a scenario where patients who failed both antimetabolites were 20% less likely to have retinal vasculitis and treatment successes were 20% more likely to have retinal vasculitis compared to the respective probabilities in those who did have FA images in this study (OR, 6.8 [95% CI, 2.4 to 19]).

**Table 2:** Crude and adjusted odds of double treatment failure compared to treatment success

**Baseline characteristic**	**Crude odds ratio** **(95% CI)**	**Adjusted odds ratio** **(95% CI)**	***P*-Value**
**Age**	0.97 (0.94, 1.01)	0.97 (0.94, 1.0)	0.086
**Sex**			
Male	Reference	Reference	
Female	1.4 (0.5, 3.8)	3.0 (0.82, 11)	0.098
**Anatomic location**			
Posterior/Panuveitis	Reference	Reference	
Anterior/Intermediate	4.5 (1.7, 12)	2.6 (0.81, 8.3)	0.11
**CME**			
No	Reference	Reference	0.15
Yes	3.4 (1.3, 8.7)	2.3 (0.74, 7.3)	
**Retinal vasculitis**			
No	Reference	Reference	
Yes	8.1 (1.8, 36)	8.6 (1.6, 47)	0.014
FA not done	3.9 (1.2, 13)	2.8 (0.77, 10)	0.12

## Discussion

While antimetabolites such as MTX and MMF are often used as first-line corticosteroid-sparing treatment for non-infectious uveitis, approximately 30-40% of patients will have inflammation that is recalcitrant to one of these therapies [7]. There is limited information to guide clinicians on the utility of trying a different antimetabolite after failing one [6], versus switching to a different class of medication altogether, such as biologics. Our study, a sub-analysis of a prospective randomized clinical trial, suggests that patients with angiographic retinal vasculitis posterior to the equator are more likely to fail both MTX and MMF.

This result is consistent with recent studies that have suggested that non-infectious retinal vasculitis may respond more effectively to biologic agents targeting tumor necrosis factor-alpha (TNF-alpha). For example, Sharma et al. examined 60 patients with retinal vasculitis who had failed other immunosuppressive therapies, and found that 88% were in remission at 6 months after being started on infliximab [9]. Another retrospective study showed that 86% of retinal vasculitis patients achieved quiescence after 12 months of therapy with either adalimumab or infliximab [10]. The most recent recommendations from the European League Against Rheumatism for the treatment of Behçets-associated uveitis, including retinal vasculitis, include anti-TNF-alpha therapies as first-line therapy [11]. Similarly, an expert panel of the American Uveitis Society recommended adalimumab and infliximab as first-line corticosteroid-sparing treatment for the ocular manifestations of Behçets [12]. Within this sub-analysis, there were a total of six patients with Behçets: two patients in the double treatment failure group and four in the treatment success group*.* Importantly, tuberculosis (TB) must be ruled out prior to starting TNF-alpha inhibitor therapy as they can increase the risk of disseminated TB. In our study, all patients had a tuberculin skin test or interferon-gamma release assay along with a chest radiograph within 90 days prior to enrollment, and patients in whom there as concern for infection were not enrolled.

The strengths of this study are the prospective, randomized, and masked collection of the data. The largest limitation of this study is that nearly 42% of patients did not have FA images obtained at baseline. The choice of obtaining an FA was based on clinical findings and suspicion for retinal vasculitis per investigator discretion and was not mandated. Other limitations include the small number of patients who failed both antimetabolites and that patients who did not continue within the trial after 6 months were not included in this sub-analysis.

## Conclusions

This study suggests that uveitis patients with retinal vasculitis are more likely to fail both MTX and MMF than patients without retinal vasculitis. Clinicians could consider advancing to other classes of corticosteroid-sparing medications, such as biologics, more quickly in these patients.

## Data Availability

The datasets used and/or analyzed during the current study are available from the corresponding author on reasonable request.

## Abbreviations

MTX: Methotrexate
MMF: Mycophenolate mofetil
FAST: First-line Antimetabolites as Steroid-sparing Treatment
CME: Cystoid macular edema
OCT: Ocular coherence tomography
FA: Fluorescein angiogram
TNF: Tumor necrosis factor
